# Evolution of infectious bronchitis virus in China over the past two decades

**DOI:** 10.1099/jgv.0.000464

**Published:** 2016-07

**Authors:** Ye Zhao, Hui Zhang, Jing Zhao, Qi Zhong, Ji-hui Jin, Guo-zhong Zhang

**Affiliations:** ^1^​ Key Laboratory of Animal Epidemiology and Zoonoses, Ministry of Agriculture, College of Veterinary Medicine, China Agricultural University, Beijing 100193, PR China; ^2^​ National Clinical Research Center of Digestive Diseases and Beijing Friendship Hospital, Capital Medical University, Beijing 100050, PR China; ^3^​ Department of Biostatistics, St. Jude Children’s Research Hospital, Memphis, TN 38105, USA

**Keywords:** infectious bronchitis virus, evolution, genotype, selection pressure

## Abstract

Avian infectious bronchitis is a highly contagious disease caused by infectious bronchitis virus (IBV) that affects poultry production worldwide. The absence of vaccine cross-protection and the frequent emergence of new variant strains complicate control of IBV. Here we designed a study to measure the evolution dynamics of IBV strains in China. One hundered and seven complete sequences and 1022 S1-region sequences of Chinese IBVs isolated between 1994 and 2014 were analysed by using MEGA 5.0 software and the Bayesian analysis sampling trees (BEAST) method, and selection pressure on different proteins was assessed. The phylogenetic dissimilarity of different gene trees in the data set indicated possible recombination. Fourteen isolates were identified as recombinants, possibly generated from vaccines of the Massachusetts serotype in recombination with circulating viruses. The earliest IBV in China was found to have existed in the early 1900s, and continues to evolve at a rate of approximately 10^−^^5^ substitutions per site per year. We found that purifying selection was the main evolutionary pressure in the protein-coding regions, while the S1 gene bears the greatest positive selection pressure. The proportion of QX-like genotype strains increased over time. These results indicate that the genotypes of Chinese IBVs have undergone a remarkable transition during the past 20 years.

## Introduction

Infectious bronchitis virus (IBV) is a coronavirus that causes acute, highly contagious, respiratory disease in poultry worldwide (Cavanagh, 2007). IBV belongs to the gamma coronaviruses, with the other two genera being mammalian viruses, including those responsible for severe acute respiratory syndrome (SARS) and Middle East respiratory syndrome (MERS) (Drexler *et al.*, 2014). The IBV genome is approximately 27.6 kb in size and contains four main structural genes, which produce the spike (S), membrane (M), envelope (E) and nucleocapsid (N) proteins.

IBV outbreaks were first reported in the USA during the 1930s; within a few years they were recognized throughout the world (Cook* et al.*, 2012). These outbreaks continue to cause great losses to the poultry industry worldwide, despite the widespread use of vaccines. Live attenuated vaccines are generally used and outbreaks are caused by the emergence of new variants, as the vaccines are not cross-protective (Cavanagh, 2007; De Wit* et al.*, 2011). Some serotypes and genotypes of IBV may remain isolated in a particular location for a period of time, while others may spread worldwide relatively rapidly.

The frequent emergence of new variant strains complicates the control of IBV. In the USA, most outbreaks are caused by the Massachusetts (Mass), Connecticut (Conn) or Arkansas (Ark) serotypes, which have been used as attenuated live vaccines. In Europe, outbreaks have been caused mainly by the 4/91 genotype and Italy-02 genotype viruses. For many years little was known about IBV variants in China. The sustained efficacy of Mass-type vaccines in China suggests that variants were not a problem there before the 1980s (De Wit* et al.*, 2011). However, since 1998 a new genotype, QX, has emerged and spread rapidly through several countries, becoming the predominant circulating genotype in that region and causing substantial economic loss (Wang* et al.*, 1998).

Vaccines have been shown to impose strong selection pressure on the evolution of circulating viruses. They may also give rise to new strains through recombination, and new genotypes may acquire greater virulence as a result of their expedited transmission in vaccinated populations. RNA viruses typically have a high rate of genomic mutation due to their low fidelity of reproduction and low processivity of their RNA polymerase, allowing the viruses to escape host defences and evolve further. The coronavirus has the largest genome of the RNA viruses, comprising nearly 28 kb. As a consequence, it is more likely to mutate under vaccination and environmental pressures (Compton* et al.*, 1993).

To understand the population dynamics of the dominant IBV genotypes in China, we investigated the evolutionary trends of the main endemic QX genotype and the Mass vaccine-like genotype in China during the 1990s–2010s by analysing a data set of 1022 complete sequences of segment 1 of the spike gene (S1) from viruses isolated at different times. The sequence comparison of 107 full-length IBV genomes, and phylogenetic and recombination analyses, were then carried out to screen out recombinant strains. We then estimated the evolution rate from a data set of full-length IBV genome sequences by Bayesian methods. Using the Lognormal relaxed uncorrelated clock method (assuming a constant rate of substitution), we estimated the earliest time of IBV circulation. We also identified the genes that have played a dominant role in IBV evolution by testing the positive selection pressure of different genes.

## Results

### Phylogenetic analysis

**Fig. 1.**
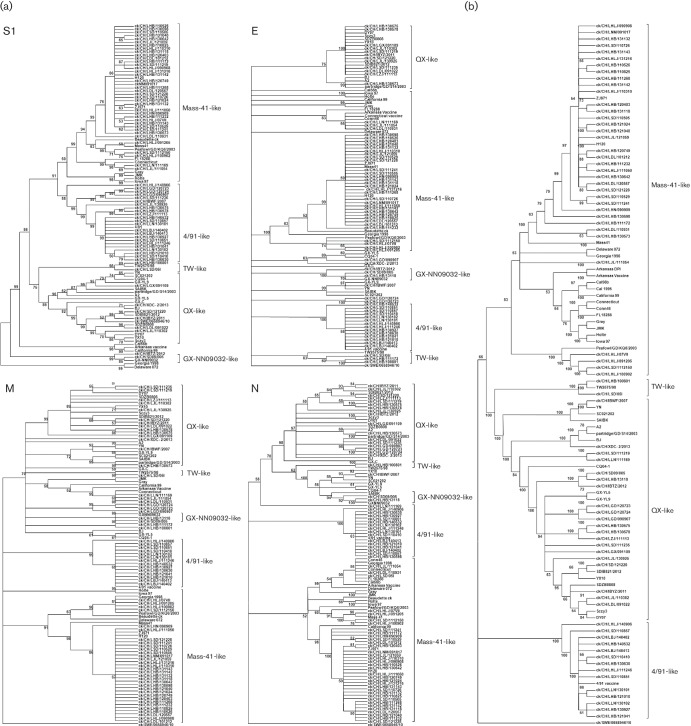
Phylogenetic relationships of different IBV genotypes. One hundred and seven different IBV isolates were involved in the phylogenetic reconstruction in mega 5. The evolutionary history was inferred using the neighbour-joining method and tested with a bootstrap test (1000 replicates). The evolutionary distances were computed using the Kimura 2-parameter method. (a) Genotype topology based on the four structural IBV genes S1, E, M and N, respectively. (b) Neighbour-joining tree inferred from entire genome sequences including untranslated regions of the 107 isolates. Bootstrap numbers are listed at the base of nodes.

The current phylogenetic classification of Chinese IBV strains is based on the full or partial nucleotide sequences of their S1, M, E or N genes. To determine whether these genes yielded consistent phylogenetic profiles, we obtained 91 full-length Chinese IBV sequences and 16 reference sequences from GenBank. We then generated nucleotide data sets for each of the four genes and a concatenated sequence of all protein-coding regions. Neighbour-joining (NJ) phylogenic trees were reconstructed for all sequence data sets under the Kimura 2-parameter model for each data set (Fig. 1). The individual and concatenated gene trees revealed several distinct genotypes, including the Mass vaccine-like group, 4/91-like group, QX-like group, Taiwan (TW)-like group, and smaller branches. The strains were not grouped entirely consistently in the different phylogenetic trees and concatenated coding sequences. To investigate the possibility of recombination among the full-length IBV sequences, we used seven different algorithms implemented in the RDP4 program. We used the strict criterion that recombinant sequences must be identified by more than five methods in the RDP4 program and have statistical support of *P*≤10^−12^ (Thor *et al.*, 2011). The different gene regions of most isolates yielded consistent phylogenic trees, while several taxa changed their genotype affiliation in different gene trees. As shown in Table 1, the recombination hotspots were mainly concentrated in the genes *1ab*, Spike and N, which have been reported to have higher mutation rates and recombination occurrence than other genes of IBV. Fourteen isolates were detected as recombinant strains based on the RDP4 screening criterion and excluded in the subsequent evolution analysis.

**Table 1.** Fourteen strains that were tested as recombinant strains by RDP4 Only transferred gene fragments with statistical support of *P *> 1×10^−12 ^are included in the table.

S1 (20371–21986)	E (24216–24542)	M (24514–25191)	N (25930–27159)	Complete genome	Recombination breakpoints	Isolate
4/91-like	QX-like	QX-like	QX-like	QX-like	17713–18631, 20474–21521	KP118886
4/91-like	QX-like	QX-like	QX-like	QX-like	5193–12258, 18632–21252	KP118889
4/91-like	QX-like	QX-like	QX-like	QX-like	5193–12258, 18632–21252	KP118890
Mass-41-like	QX-like	QX-like	QX-like	Mass-41-like	902–18504, 20371–21627	KJ425496
Mass-41-like	Mass-41-like	Mass-41-like	4/91-like	Mass-41-like	5916–9079, 19872–21262	KJ425497
4/91-like	QX-like	QX-like	QX-like	QX-like	15023–17670, 17698–21317	KP118894
4/91-like	QX-like	QX-like	QX-like	QX-like	4840–7515, 17704–23235	KP036505
Mass-41-like	TW-like	TW-like	Mass-41-like	Mass-41-like	23575–24551	KJ425489
GX-NN09032-like	QX-like	QX-like	QX-like	QX-like	18842–24420	KF663559
4/91-like	QX-like	QX-like	QX-like	QX-like	6714–8599, 20445–21128	KF663560
GX-NN09032-like	QX-like	QX-like	QX-like	QX-like	19637–23546	KF668605
Mass-41-like	Mass-41-like	Mass-41-like	QX-like	QX-like	24552–26167	KF411040
4/91-like	QX-like	QX-like	QX-like	QX-like	16207–17648, 18829–21252	JX195176
TW-like	TW-like	Mass-41-like	Mass-41-like	TW-like	4582–22625, 24013–26178	EU637854

### Estimating ancestral dates using the molecular clock method

**Fig. 2.**
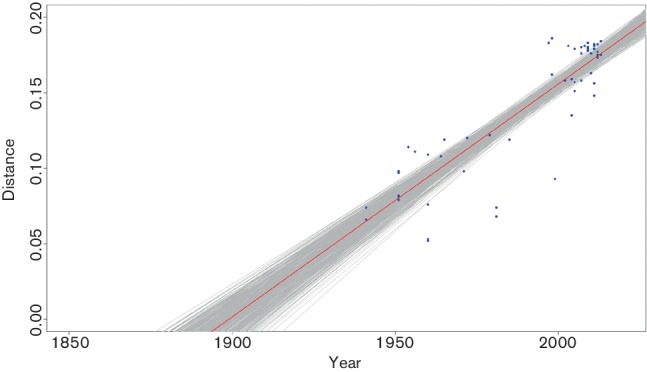
Time of the last common ancestor of IBV. Calculations were based on the branch length of the phylogenetic tree of the entire genome sequences including UTRs of the 107 isolates. The branch length (distance) from each cluster to the root of the tree is plotted against time (year) for each sequence (blue dots). A maximum-likelihood linear fit (bold red line) was generated as described in Methods. The 95 % CIs (grey area encompassing red line) were calculated by 480 bootstrap fits to data points.

The branch length from each cluster to the root of the tree was plotted against time for the 91 complete sequences of Chinese isolates showed in Table S1 (available in the online Supplementary Material), and a maximum-likelihood linear fit was generated. The results, assuming a strict molecular clock (a constant rate of substitution), suggested that the most recent common ancestor in China existed near 1900. The 95 % confidence intervals (CIs) roughly spanned the last 20 years of the nineteenth century through the first 20 years of the twentieth century (1882.4–1919.5) (Fig. 2).

### Evolutionary rates of different genes in Chinese IBVs

We inferred the evolutionary rates of Chinese IBV strains whose date of isolation was known, by using a Bayesian coalescent approach based on the sequence data sets of different protein-coding genes (excluding the 14 recombinant sequences). Under the best-fit model, Bayesian estimates of the mean substitution rates of these genes were between 2.43×10^–5^ and 9.77×10^–5^ substitutions per site per year (Table 2). The E gene evolved at the fastest rate among the structural protein–encoding genes, with a substitution rate of 9.77×10^–5^ and a highest probability density (HPD) of between 2.45×10^–5^ and 1.79×10^–4^. The N gene evolved at the slowest estimated mean (95 % HPD) rate of 2.43×10^–5^ (1.61×10^−8^–5.02×10^–5^).

**Table 2.** Bayesian estimates of evolutionary rate of specific protein-encoding genes of IBV

Gene	Evolutionary rate (nt substitutions per site per year)	95 % HPD
S1	2.93×10^−^^5^	4.61×10^−^^6^–5.71×10^−^^5^
E	9.77×10^−5^	2.45×10^−5^–1.79×10^−4^
M	6.60×10^−^^5^	3.22×10^−^^6^–1.35×10^−4^
N	2.43×10^−^^5^	1.62×10^−^^8^–5.02×10^−^^5^

HPD, highest probability density.

### Positive selection of different genes in Chinese IBVs

To identify additional forces affecting IBV evolution, we analysed the average selective pressure per protein by using Model 0 (M0) of the codon-based phylogenetic models (codeml) within the phylogenetic analysis by maximum-likelihood (paml) programs. This assumes a constant substitution rate at all sites and provides estimated values averaged across all sites. The gene encoding the S1 protein was found to be most variable, while the gene that encodes non-structural 1ab protein was more conserved. Based on Bayesian analyses, neutral and positive selection models were compared by using likelihood ratio tests. The neutral models (M0, M1 and M7) with a proportion of selected codons (M2 and M8), and a model for difference between non-synonymous (dN) and synonymous (dS) rates (dN/dS)among amino acid residues (M3), were applied to test the selective pressure on different protein-encoding residues (Table 3). The log likelihood values indicated that positive selection models (M2, M3, M8) fitted most of the gene regions tested better than neutral models (M0, M1, M7). The nested comparisons between neutral and positive models, including M0 versus M3, M1 versus M2 and M7 versus M8, confirmed the better fit of positive models, suggesting that positive selection occurs at different gene sites within the genome during the evolution of IBV. The number of protein-coding sites within the S1 glycoprotein–coding region under positive selection pressure was most among the different gene regions (*P *< 0.05 in these three comparisons, chi-square test) (Table 3).

**Table 3.** Amino acid sites under positive selection pressure and likelihood ratio tests comparing positive selection models vs neutral models for different IBV gene segments Positive selection models: M2a, M3, M8; neutral models: M0, M1a, M7. Positive selection pressure was defined as dN/dS > 1, where dN = number of non-synonymous substitutions per site and dS = number of synonymous substitutions per site. Positive selection site was determined by probability in Bayes empirical Bayes (BEB) analysis. ω, dN/dS ratio; L, log likelihood; M0, one ratio model; M1, nearly neutral model; M2, positive selection model; M3, discrete model; M7, beta model; M8, beta and ω model. na, Not analyzed; nf, not found. *P≤0.05; **P≤0.01.

Protein	M0 (*ω*)	Likelihood test (2△LnL)			Positive selection site (aa)
		M0 vs M3	M1a vs M2a	M7 vs M8	
S1	0.37080	2243.276376**	277.436402**	306.1185**	19L; 23K; 57T; 64S; 65D; 72Y; 96P; 98A; 101S; 105A; 130Q; 132S; 141Q; 174K; 301S; 307S; 316Q; 357N; 406R; 409T; 410R; 417R; 421T; 550F; 566S
E	0.19028	89.030374**	0.369408	8.355172*	3L; 25V; 79S
M	0.19238	331.734492**	10.425006**	51.90527**	5E; 12S; 47L
N	0.17251	754.57614**	40.22616**	60.60845**	64S; 300R; 337P
3a	0.44618	40.87654**	1.05086	2.37579	nf
3b	0.41865	78.455836**	10.71828**	11.38464**	nf
5a	0.30974	97.822628**	5.329922	19.34628**	61D
5b	0.60993	152.690428**	29.47067**	31.11947**	23E; 39P; 57I; 58D; 73S; 81S
Nsp2	0.19536	462.915408**	0	na	nf
Nsp3	0.15092	145.411142**	0	1.962352	264N
Nsp4	0.16252	506.51592**	0	17.12271**	8W; 387T; 456A; 498I
Nsp5	0.16082	423.24087**	21.774806**	26.65132**	66T; 177T; 221L
Nsp6	0.15092	145.411142**	0	1.962352	nf
Nsp7	0.11175	6.597726	0.000714	0.098472	nf
Nsp8	0.14546	101.37074**	0	14.46742**	nf
Nsp9	0.09007	31.338902**	0.010186	6.5499*	nf
Nsp10	0.06749	85.882382**	0	10.37397**	nf
Nsp12	0.06939	458.716066**	17.618464**	70.96948**	22Q; 45C; 64A
Nsp13	0.06920	183.729002**	0.000162	5.99636	nf
Nsp14	0.07150	272.803576**	0.061184	8.457994*	nf
Nsp15	0.09815	303.64197**	0	4.891118	nf
Nsp16	0.09701	484.788486**	6.245764*	32.88781**	209I; 275F; 294T; 297S

### Population dynamics of IBV genotypes over the past 20 years

**Fig. 3.**
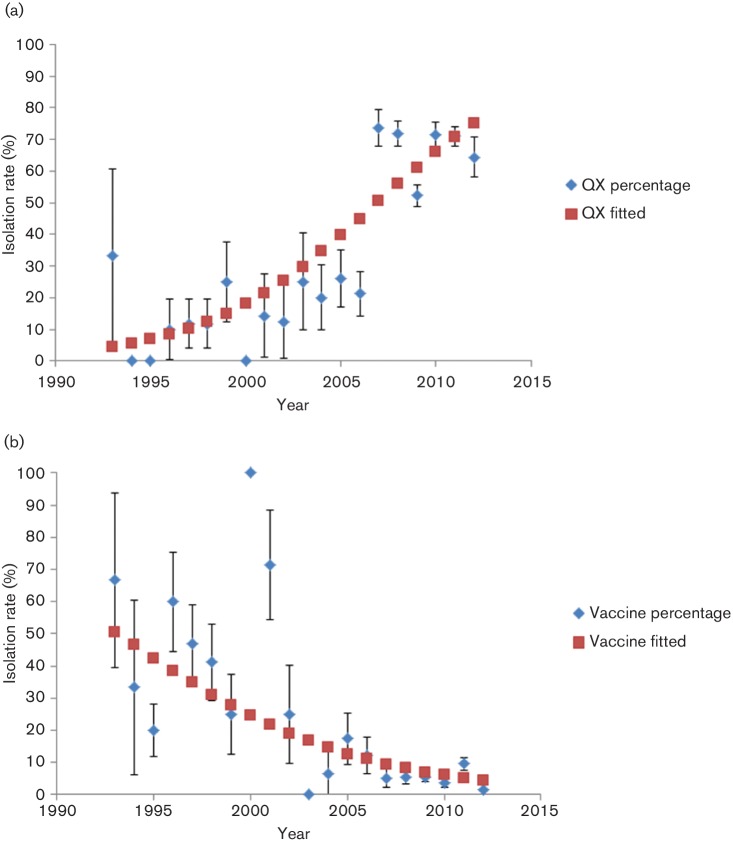
Population dynamics of QX-like and vaccine (Mass)-like genotype IBVs during the past 20 years. The proportion of QX-like (a) and vaccine-like (b) sequences was calculated from the 1022 strains isolated each year (blue diamonds). A logistic regression was performed to model the isolation rate over time, and the model fitted is shown as a line with red squares.

In order to understand the composition change of the IBV strains circulating in China over the past 20 years, we collected 1022 S1 gene sequences of Chinese IBVs (collected during 1994–2014) from GenBank. These S1 sequences were divided into eight genotypes based on their phylogenetic trees. These comprised (1) a vaccine-like group comprising common vaccine strains (Mass 41, H120, Cal99, CU-T2, Gray, Conn and Ark) and field isolates; (2) a 4/91-like group including UK 793 and other field isolates; (3) a TW-like group represented by TW2575/98, TW97-4 and TP/64; and (4) five main Chinese groups (BJ-like, QX-like, CK/CH/LSC/95I-like, GX-like and GM-like). Most of the isolates clustered into genotype QX. To investigate the change of isolation rate over time, a single-covariate logistic regression was performed using isolation as response and year as covariate. As time elapsed, we saw a sustained increase in the QX-like genotype in China (from 11.7 % to nearly 70 % at present) (Fig. 3), while the proportion of the vaccine-like genotype was implied to decline from 50.4 % to 4.4 % from the mid-1990s to the present (Fig. 3). This result suggests that the genotypes of Chinese IBVs have undergone a remarkable transition during the past 20 years.

## Discussion

Our study of the evolution of Chinese IBV strains, using available full-length genome sequences and S1 protein-coding sequences, demonstrated an inconsistency between genotype affiliations based on individual genes versus concatenated full-length genomes, indicating that recombination events exist widely in the IBV populations and influence the topology of phylogenetic trees. The S1 gene tree was closest to the complete-genome phylogenetic tree. The S1 gene is known to be the main antigenic determinant of IBV (Cavanagh & Naqi, 2003) and is used as the conventional genotyping evidence of IBV (Lee* et al.*, 2003; Wang & Huang, 2000). However, in several strains the phylogenetic trees of some genes showed a change in their genotype affiliations, suggesting recombination between different circulating IBV strains. Further, 14 sequences showed the possibility of recombination of different genotypes. Among the 14 recombinants we identified, most had a major parent of the QX type, with minor parents of the Mass-41-like, 4/91-like, TW-like or Ark-like genotypes. Our identification of recombinants derived from Mass-41-like vaccine strains confirms that chickens can be simultaneously infected with live virus vaccine and other circulating IBV genotypes (Kottier* et al.*, 1995). Therefore, incorrect vaccination strategies could inadvertently lead to the mixture of vaccine strains and WT virus. There have been many reports of recombination between other vaccine strains and circulating wild strains, including a PMV-1 (Chong* et al.*, 2010), bovine viral diarrhea virus (Becher* et al.*, 2001) and infectious bursal disease virus (Hon* et al.*, 2008), raising concern that modified live virus vaccines, although efficacious, may facilitate the emergence of new strains through recombination with circulating viruses.

In most instances, it is less important to know the isolation date than the location of isolated sequences. However, in the case of fast-evolving organisms such as RNA viruses, changes in the date of isolation can be used to estimate the time since viruses last shared a common ancestor. Therefore, analyses based on the assumption of a constant rate of molecular evolution, like those used to estimate the date of divergence between sequences, require the date of isolation. More importantly, the differences in isolation dates, under the assumption of rate constancy, provide information about the rate of molecular evolution. That is, the quantity of evolutionary change that has accumulated since the date of isolation (Thorne* et al.*, 1998). The first outbreaks of IBV in the USA were reported during the 1930s, which is consistent with the results inferred through full-length sequences in this study assuming a strict molecular clock. This method has been used successfully to determine the timing of ancestors of the human immunodeficiency virus 1 (HIV-1) pandemic strains (Korber* et al.*, 2000) and SARS virus (Zeng* et al.*, 2003). Our results indicate that the most recent ancestor of IBV existed in the early 1900s, indicating that IBV may have been circulating in nature for a period before its first outbreak. A Bayesian coalescent approach indicated a mean substitution rate of between 2.43×10^–5^ and 9.77×10^–5^ substitutions per site per year in different genes, which is comparable with the results reported for hepatitis A (Wang* et al.*, 2015) and B (Alvarado Mora* et al.*, 2011) virus and low-virulence Newcastle disease virus (Miller* et al.*, 2009). The higher substitution rate of the IBV E region may be due to its less important function in virus synthesis and replication than other regions, which may have been subjected to greater immune selection pressure and undergone more substitutions.

When we compared the selection profiles of protein-encoding genes of Chinese IBVs, the global rate of non-synonymous to synonymous substitutions (dN/dS) in all protein-coding genes was less than 1, indicating purifying selection as the major force driving the evolution of Chinese IBV viruses. However, there was a clear difference in the selection profiles of different genes. The S1 gene had the highest dN/dS value among the main structural protein-coding genes. The 25 protein-coding sites within the S1 glycoprotein coding region were detectably evolving under positive selection pressure. Frequent point mutations in the hypervariable regions of the spike 1 (S1) gene contributed to most of the antigenic determinants of IBV. The S protein is essential to induce protective immunity, and small differences in the S gene contribute to poor cross-protection. As the S1 subunit of the spike protein is the major antigenic moiety of coronaviruses and is not an essential structural protein, it is prone to high mutation rates as the virus evolves in host populations. While the IBV N protein has generally been conserved and negatively selected for during viral evolution, we identified three positively selected sites at aa 64S, 300R and 337P in the N protein of IBV, consistent with a previous study (Kuo* et al.*, 2013). The 64S site has been demonstrated to be located in the putative recombinant region of the NTD domain and is critical for binding to TRS repeats (Kuo* et al.*, 2013). In addition, it has been suggested that the α-turn region (aa 63–67) may form an antigenic epitope in the IBV N protein (Ignjatovic & Sapats, 2005). The other two sites have not been included in other studies but may also be important in the N protein for coronaviral RNA binding. Among the non-structural protein coding genes, *3a*, *3b*, *5a* and *5b* bear greater positive selection pressure than does gene *1* (Nsp2-16). Gene 1 comprises approximately two-thirds of the genome and encodes two overlapping ORFs, *1a* and *1b*, which are translated as the large polyprotein 1ab. Protein 1ab can be divided into 15 or 16 non-structural proteins and is associated with RNA replication and transcription (Lai & Cavanagh, 1997). Viral fitness can indeed limit the variability of genes, which is critical to virus replication (Söderholm* et al.*, 2006). Thus, it is reasonable that gene *1* undergoes mainly purifying selection in the evolution process of IBV. Our data support the conclusion that positive selection has occurred in most gene parts of Chinese IBV. This selection may promote viral fitness in infected hosts.

The population dynamics of the QX-like and Mass (vaccine)-like Chinese IBV isolates show that the proportion of QX-like isolates has undergone a sustained increase during the past 20 years, while the population of vaccine-like genotypes is inferred to have declined since the mid-1990s. Since the QX-like genotype was first reported in China in 1998, it has rapidly increased worldwide and is now the predominant IBV genotype. During the past few years, the QX-like viruses have made up a great proportion of strains identified in many epidemiological investigations worldwide. The poultry industry has, in recent years, detected an increasing incidence of outbreaks of QX-like IBV strains in many countries. Despite the widespread use of live attenuated IBV vaccines (Mass serotype), such as strains H120, H52 and Ma5, vaccinated chicken flocks usually fail to obtain complete protection against challenge with virulent field IBV. Furthermore, we previously found that the virulence of Chinese IBV isolates is increasing (Feng* et al.*, 2012). The genotype misfit of vaccine and wild viruses results in the sustaining of multiple virulent strains and more complicated situations. Thus, optimal vaccines against circulating IBV strains in China should be attenuated viruses developed against local strains.

In conclusion, our results indicate that Chinese IBV isolates are evolving at a rapid rate under the immune selection pressure of vaccines. Therefore, it is possible that the avian immune system has reshaped the IBV genotype and phenotype through positive selection, although genetic mutation and recombination contribute to the natural evolution of IBV. The S1 gene is the first structural gene to be affected by such selection pressure to our knowledge. As the proportion of QX-like isolates becomes larger over time, there will be a greater need for vaccines with new genotypes.

## Methods

### IBV strains.

We downloaded 107 complete genome sequences of IBV strains obtained before 2015 from GenBank. To estimate rates of nucleotide substitution per site per year and the time of appearance of the earliest IBV isolate, we included only sequences with a known year of collection. Dates of collection for each sequence were determined either from GenBank annotations or the journal article describing the sequence. Detailed information on the IBV strains analysed is provided in Table S1, including the GenBank accession number, year of isolation and site of isolation (in the absence of specific location, the site is listed as China). Different data sets, including the sequences of the S1, M, E and N structural genes and the non-structural genes, were extracted from the complete genome data and used in analyses. An additional 1022 IBV S1 gene sequences, mostly isolated from China, were collected from GenBank to calculate the changes in percentage of different genotypes in China over the past 20 years; the GenBank accession numbers of these strains are provided in Table S2.

### Phylogenetic analysis.

Partial nucleotide sequence data sets of the S1, M, E and N genes were extracted from the 107 complete genome sequences, while the 1022 full-length S1 genes were stored in a separate data set. Multiple sequence alignment of all sequence data sets was performed using the codon-based Clustal W program. The DNA sequences were translated into amino acid sequences by using the software dambe 4.5.20. Maximum-likelihood phylogenic trees were reconstructed by using the appropriate nucleotide substitution model selected for each data set using mega 5.0 software (Tamura* et al.*, 2007). Results were validated by 1000 bootstrap replicates.

Undetected recombination events can muddle the evolutionary relationships in the tree (Kusters* et al.*, 1990). To remove sequences that had undergone possible recombination from subsequent evolution analysis, searches for recombinant sequences and crossover regions were performed by aligning the 107 full-length sequences in the Geneconv (Sawyer, 1999), RDP (Maidak* et al.*, 1997; Martin* et al.*, 2010), MaxChi (Smith, 1992), Chimera (Posada & Crandall, 2001), BootScan (Martin* et al.*, 2005), SiScan (Gibbs* et al.*, 2000), 3Seq (Sled* et al.*, 1998) and LARD (Holmes* et al.*, 1999) programs, all implemented in Recombination Detection Program v.4.16 (Martin* et al.*, 2010). Programs were executed with modified parameter settings according to the guidelines in the RDP4 manual for the analysis of divergent sequences (Martin* et al.*, 2015). Recombinant sequences were tested with the highest acceptable *P*-value of 0.05. To eliminate interference from known recombinant isolates, we excluded recombinant isolates identified in other studies.

### Evolution substitution rates.

We analysed 107 representative complete genome sequences of IBV isolated between 1941 and 2014 by using the MCMC program in the beast software package (version 1.8.2) to estimate the rate of molecular evolution of different genes. The program provides a maximum-likelihood estimate of the rate, under a model that assumes a constant rate of substitution (molecular clock). Confidence intervals for these parameters were estimated.

jModel Test software 2.0.1 was used (Posada, 2008) to estimate the best-fit model according to the Akaike information criterion (AIC). The Bayes factor was used for model comparison to determine which model yielded the best results. This factor was calculated for each model tested, using marginal likelihood estimated according to the method of Newton & Raftery (1994), with the modifications proposed by Drummond *et al. *(2012). GTR (general time reversible) + I (proportion of invariant sites) and Γ4 (gamma distributed rate variation with four rate categories) + Lognormal relaxed uncorrelated clock with expon-growth demographic population dynamic models, as implemented in the beast package, was found to be the best-fit model for this study. The MCMC analysis was performed with 100 million generations and was sampled every 10 000 generations with 10 % burn-in. The unweighted pair group method with arithmetic mean was used to reconstruct a starting tree. The results were computed and analysed by using the Tracer 1.6 program. The effective sample size was over 200 for estimation of parameters in the MCMC analysis. Statistical uncertainty in the data was reflected in the 95 % HPD values.

### Estimated time of IBV emergence in China. 

Given a phylogenetic tree and assuming a uniform rate of evolution, we can plot total branch length (from branch tip to ancestral node) against the year of sampling and fit a line through the data points (Korber* et al.*, 2000). From this inferred linear relation, we can project back to estimate the time of zero branch length. For a standard linear least-squares fit to a line, one implicitly assumes that the data on the independent axis (sampling time) are precisely known, and the best-fit line is chosen to minimize the squared deviation on the dependent axis (branch length). Bootstrap re-sampling of the data points (480 trials) provided 95 % CIs for estimated timing of ancestral sequences (Fig. 2).

### Positive selection analysis. 

Variable selective pressures at individual codon positions of different proteins were evaluated on the basis of phylogenetic analysis of the complete genome of 107 IBVs. We applied paired models of variable dN/dS distribution among amino acid sites, including M3 (discrete) versus M0 (one ratio), M2 (positive selection) versus M1 (nearly neutral) and M8 (beta and dN/dS) versus M7 (beta), in the codon-based phylogenetic models (codeml) program within the paml software package. The likelihood ratio test statistic was calculated as twice the log likelihood (L) difference between the two models and labelled 2 (L1–L0). Values were compared by chi-square test with one degree of freedom (equal to the difference between the compared models in the number of free parameters).

### Proportional change of the genotypes of IBV isolate over time.

We compiled the 1022 S1 gene sequences of Chinese IBV strains isolated from 1994 to 2014 and analysed these sequences by using mega 5.0. Phylogenetic trees were reconstructed by using the maximum-likelihood method with 1000 bootstrap replicates. The sequences were divided into different genotypes on the basis of this phylogenetic tree and the number of isolates in different groups was summed, respectively, to calculate the percentages of the respective genotypes. The variation trend line of the number of QX-like and vaccine (Mass)-like groups was reconstructed by using statistical methods.

## Supplementary Data

422 Supplementary File 1
