# Supplementary material for: Evolution of infectious bronchitis virus in China over the past two decades
Source: J Gen Virol. 2016 Jul;97(7):1566–74. doi: 10.1099/jgv.0.000464 (PMC7079583; doi:10.1099/jgv.0.000464)
Supplement: Supplementary file 1 [file jgv-97-1566-s001.pdf]

Supplementary Table 1. 107 complete genome sequences of infectious bronchitis virus strains included in the study

Accession number	Full virus Names	Date isolated	Isolation site	Length (nt)
AY851295	Mass41	1941	USA	27475
GU393336	Holte	1954	USA	27247
GU393337	Iowa 97	1956	USA	27663
GU393334	Gray	1960	USA	27622
EU817497	H52	1960	Netherlands	27646
FJ888351	H120	1960	Netherlands	27652
GU393338	JMK	1964	USA	27515
GU393333	FL18288	1971	USA	27616
GQ504721	Arkansas Vaccine	1981	USA	27620
KF377577	4/91 vaccine	1991	UK	27618
GU393331	Cal56b	1991	USA	27663
GU393332	Delaware 072	1992	USA	27591
GQ504723	Georgia 1998 Vaccine	1998	USA	27620
AY514485	California 99	1999	USA	27693
JQ088078	ck/SWE/0658946/10	2010	Sweden	27664
KF696629	Connecticut vaccine	1951	USA	27630
KC008600	GX-C	1985	China	27701
EU526388	A2	1996	Beijing	27715
EU714028	ZJ971	1997	Zhejiang	27640
DQ646405	TW2575/98	1998	Taiwan	27710
DQ288927	SAIBK	2000	Sichuan	27534
EU714029	SC021202	2002	Sichuan	27660
AY646283	partridge/GD/S14/2003	2003	Guangdong	27503
AY641576	Peafowl/GD/KQ6/2003	2003	Guangdong	27434
AY319651	BJ	2003	Beijing	27733
HM245924	CQ04-1	2004	Chongqing	27709
JF893452	YN	2005	Yunnan	27635
HQ848267	GX-YL5	2005	Guangxi	27720
EU637854	ck/CH/LSD/05I	2005	Shandong	27638
KF663560	ck/CH/IBWF/2007	2007	China	27661
JF274479	ck/CH/LHLJ/07VII	2007	Heilongjiang	27678
HQ850618	GX-YL9	2007	Guangxi	27597
HM245923	DY07	2007	Sichuan	27675
KJ524708	SDZB0808	2008	Shandong	27677
KJ425503	ck/CH/LHLJ/090908	2009	Heilongjiang	27609

KJ425504	ck/CH/LHLJ/091205	2009	Heilongjiang	27485
KJ425508	ck/CH/LHN/090909	2009	Henan	27641
KF668605	ck/CH/SD09/005	2009	Shandong	27691
KF411041	ck/CH/LGX/091109	2009	Guangxi	27655
JX897900	GX-NN09032	2009	Guangxi	27684
JX195175	ck/CH/LDL/091022	2009	Dalian	27685
JF732903	Sczy3	2009	Sichuan	27695
KP118894	ck/CH/LGD/090907	2009	Guangdong	27685
JF330899	ck/CH/LNM/091017	2009	Neimenggu	27631
JX840411	YX10	2010	Zhejiang	27674
JF828980	ck/CH/LHLJ/100902	2010	Heilongjiang	27485
JF828981	ck/CH/LDL/101212	2010	Dalian	27641
JF330898	ck/CH/LHB/100801	2010	Hebei	27675
KP118884	ck/CH/LSD/110851	2011	Shandong	27617
KP118885	ck/CH/LSD/110857	2011	Shandong	27618
KP118886	ck/CH/LSD/111235	2011	Shandong	27686
KP118891	ck/CH/LHLJ/111246	2011	Heilongjiang	27618
KP118893	ck/CH/LSD/110410	2011	Shandong	27618
KJ425485	ck/CH/LDL/110931	2011	Dalian	27594
KJ425487	ck/CH/LHB/110526	2011	Hebei	27642
KJ425488	ck/CH/LHB/110825	2011	Hebei	27642
KJ425489	ck/CH/LHB/111172	2011	Hebei	27642
KJ425490	ck/CH/LHB/111232	2011	Hebei	27641
KJ425491	ck/CH/LHB/111268	2011	Hebei	27642
KF411040	ck/CH/LLN/111169	2011	Liaoning	27663
KJ425505	ck/CH/LHLJ/110310	2011	Heilongjiang	27642
KJ425506	ck/CH/LHLJ/111050	2011	Heilongjiang	27642
KJ425510	ck/CH/LSD/110505	2011	Shandong	27642
KJ425511	ck/CH/LSD/110529	2011	Shandong	27642
KJ425512	ck/CH/LSD/110726	2011	Shandong	27642
KJ435283	ck/CH/LSD/111219	2011	Shandong	27666
KJ435284	ck/CH/LSD/111241	2011	Shandong	27616
KJ435286	ck/CH/LSD/1112150	2011	Shandong	27485
KF663561	ck/CH/IBYZ/2011	2011	China	27673
KC506155	ck/CH/LJL/111054	2011	Jilin	27648
KC136209	ck/CH/LJL/110302	2011	Jilin	27685
JX195176	ck/CH/LZJ/111113	2011	Zhejiang	27683
KJ425486	ck/CH/LDL/120557	2012	Dalian	27644
KJ425492	ck/CH/LHB/120403	2012	Hebei	27642
KJ425493	ck/CH/LHB/120749	2012	Hebei	27642

KJ425494	ck/CH/LHB/121024	2012	Hebei	27641
KJ425495	ck/CH/LHB/121040	2012	Hebei	27642
KJ425509	ck/CH/LJL/121059	2012	Jilin	27642
KJ435285	ck/CH/LSD/121228	2012	Shandong	27643
KJ128295	ck/CH/SD/121220	2012	Shandong	27666
KF663559	ck/CH/IBTZ/2012	2012	China	27700
KF574761	SDIB821/2012	2012	Shandong	27677
KC013541	ck/CH/LGD/120723	2012	China	27718
KC119407	ck/CH/LGD/120724	2012	China	27718
KP036503	ck/CH/LHB/121010	2012	Hebei	27613
KP118883	ck/CH/LHB/121041	2012	Hebei	27615
KP118888	ck/CH/LLN/130102	2013	Liaoning	27617
KP118889	ck/CH/LHB/130575	2013	Hebei	27682
KP118890	ck/CH/LHB/130578	2013	Hebei	27682
KP118892	ck/CH/LLN/130101	2013	Liaoning	27594
KM213963	ck/CH/XDC-2/2013	2013	China	27714
KJ425496	ck/CH/LHB/130573	2013	Hebei	27606
KJ425497	ck/CH/LHB/130598	2013	Hebei	27621
KJ425498	ck/CH/LHB/130642	2013	Hebei	27642
KJ425499	ck/CH/LHB/131118	2013	Hebei	27642
KJ425500	ck/CH/LHB/131132	2013	Hebei	27644
KJ425501	ck/CH/LHB/131142	2013	Hebei	27642
KJ425502	ck/CH/LHB/131143	2013	Hebei	27642
KJ425507	ck/CH/LHLJ/131216	2013	Heilongjiang	27642
KM213963	ck/CH/XDC-2/2013	2013	China	27714
KP036504	ck/CH/LHB/130630	2013	Hebei	27617
KP036505	ck/CH/LJL/130925	2013	Jilin	27676
KP118880	ck/CH/LHB/130927	2013	Hebei	27618
KP118887	ck/CH/LHB/140532	2014	Hebei	27621
KP036502	ck/CH/LHLJ/140906	2014	Heilongjiang	27611
KP118881	ck/CH/LBJ/140413	2014	Beijing	27618
KP118882	ck/CH/LBJ/140402	2014	Beijing	27618

Accession numbers are from GenBank. “China” is listed where no specific site is known. Chinese sites are province or city names. USA, United States of America; UK, United Kingdom. M41, H52, H120, Arkansas Vaccine, 4/91 vaccine, Georgia 1998 Vaccine and Connecticut vaccine are all attenuated strains.

Supplementary Table 2. 1022 S1 sequences of infectious bronchitis virus strains included in the study

Accession number	Full virus Names	Accession number	Full virus Names
KJ873875	HN_LH	KJ524616	ck/CH/GX/GL12-2
KJ524580	ck/CH/GD/LZ11	KJ524617	ck/CH/YN/SL11-1
KJ524581	ck/CH/GD/HY11	KJ524618	ck/CH/YN/SL11-2
KJ524582	ck/CH/GX/NN11-1	KJ524619	ck/CH/GX/GL12-3
KJ524583	ck/CH/GX/NN11-2	KJ524620	ck/CH/YN/SL12-1
KJ524584	ck/CH/GX/GL11-1	KJ524621	ck/CH/ZJ/HZ12
KJ524585	ck/CH/GX/GL11-2	KJ524622	K/CH/SC/DY12-3
KJ524586	ck/CH/FJ/PT11	KJ524623	ck/CH/GX/NN12-1
KJ524587	ck/CH/JS/YC11-1	KJ524624	ck/CH/GX/ZS12
KJ524588	ck/CH/JS/YC11-2	KJ524625	ck/CH/HuN/NX12
KJ524589	ck/CH/HuB/HC11	KJ524626	ck/CH/SC/MS12-1
KJ524590	ck/CH/GD/YN11	KJ524627	ck/CH/SC/MS12-2
KJ524591	ck/CH/GD/LD11	KJ524628	ck/CH/JS/LYG12
KJ524592	ck/CH/GD/CG11	KJ524629	ck/CH/HuB/HC12
KJ524593	ck/CH/SC/MS11-1	KJ524630	ck/CH/FJ/ZZ12
KJ524594	ck/CH/SC/MS11-2	KJ524631	ck/CH/YN/SL12-2
KJ524595	ck/CH/SC/ZJ11	KJ524632	ck/CH/YN/SL12
KJ524596	ck/CH/GX/LC11-1	KJ524633	ck/CH/YN/SL12-4
KJ524597	ck/CH/GX/LC11-2	KJ524634	ck/CH/GX/NN12-2
KJ524598	ck/CH/GX/LC11-3	KJ524635	ck/CH/GX/GL12-4
KJ524599	ck/CH/GX/NN11-3	KJ524636	ck/CH/YN/SL12-3
KJ524600	ck/CH/SC/MS11-3	KJ524637	ck/CH/HuB/XN11
KJ524601	ck/CH/SC/MS11-4	KJ524638	ck/CH/GD/LZ12
KJ524602	ck/CH/GX/NN11-4	KJ524639	ck/CH/HuN/CX12
KJ524603	ck/CH/AH/WH11	KJ524640	ck/CH/GX/YL12-2
KJ524604	ck/CH/ZJ/QZ11	KJ524641	ck/CH/GD/XX12
KJ524605	ck/CH/SC/DY11	KJ544558	ck/CH/FJ/PT12
KJ524606	ck/CH/SC/MS11-5	KC478646	ck/CH/Shaanxi/2009/W09
KJ524607	ck/CH/GX/GL11-4	KC478647	ck/CH/Shaanxi/2009/H09
KJ524608	ck/CH/GX/YL12-1	KC478648	ck/CH/Shaanxi/2012/WN
KJ524609	ck/CH/GD/CG12-2	KC478649	ck/CH/Shaanxi/2012/BJ
KJ524610	ck/CH/GD/CG12-1	KF007201	SDIB662/2011
KJ524611	ck/CH/ZJ/QZ12-1	KF007202	SDIB669/2012
KJ524612	ck/CH/ZJ/QZ12-2	KF007203	SDIB679/2012
KJ524613	ck/CH/SC/DY12-1	KF007204	SDIB702/2012
KJ524614	ck/CH/SC/DY12-2	KF007205	SDIB768/2012
KJ524615	ck/CH/GD/LY12	KF007206	SDIB763/2012
JQ764815	GX-C	KF007207	SDIB764/2012
JQ764816	GX-G	KF007208	SDIB778/2012

JQ764817	GX-LZ1	KF007209	SDIB781/2012
JQ764818	GX-NN6	KF007210	SDIB821/2012
JQ764819	GX-NN8	KC577387	45HeB-96I
JQ764820	GX-NN10	KC577388	48SD-96VI
JQ764821	GX-NN12	KC577389	49AH-97I
JQ764822	GX-YL1	KC577390	50HeN-99III
JQ764823	GX-YL2	KC577391	53XJ-99II
JQ764824	GX-YL6	KC577392	54HaN-95I
JQ764825	GX-YL7	KC577393	55HaN-95II
JQ764826	GX-YL9	KC577394	56GX-98I
JQ764827	GX-NN5	KC577395	58HeN-93II
JQ764828	GX-XD	KC577396	60SC-93II
KC795604	QX-IBV	KC577397	66GD-98VI
KC708008	ck/CH/FNO-E/2009	KC577398	68GX-09I
KC577362	11HIBV97	KC577399	69GX-08I
KC577363	12SWIBV97	KC577400	70GX-08II
KC577364	13JNIBV98	KC577401	72HaN-09I
KC577365	QXIBV2	KC577402	73GX-08III
KC577366	16GNIBV98	KC577403	74GD-09I
KC577367	17HeBIBV98	KC577404	75GX-07I
KC577368	18HeNIBV98	KC577405	78GX-08IV
KC577369	19JSNJ97	KC577406	79HaN-09III
KC577370	24SDPL-01I	KC577407	80GX-09IV
KC577371	25SDZC-01I	KC577408	81HaN-09IV
KC577372	26SDTA-01I	KC577409	83GX-09V
KC577373	27SDTA-01II	KC577410	84CQ-09I
KC577374	30JX-99I	KC577411	87GX-08VII
KC577375	31JL-97I	KC577412	88GX-08VIII
KC577376	32JS-97I	KC577413	89HaN-09VI
KC577377	33JS-95III	KC577414	90GX-08IX
KC577378	34TJ-96II	KC577415	91GX-08X
KC577379	35SD-97I	KC577416	93GD-09II
KC577380	36SD-97II	KC577417	95GD-09III
KC577381	37HeN-96IV	KC577418	96GX-09VI
KC577382	40GDGZ-97I	KC577419	97HaN-09VIII
KC577383	41SD-97IV	KC577420	98GD-09IV
KC577384	42HLJ-98I	KC577421	101GD-10I
KC577385	43SD-96III	KC577422	102GD-10II
KC577386	44SX-96I	KC577423	103GD-10III
KC577424	104GD-10IV	JX291987	GX-NN-9
JX291983	GX-NN-4	JX291985	GX-NN-6
JX291984	GX-NN-5	JX291986	GX-NN-8
JX291988	GX-NN-11	KC357729	HC13
JX291989	GX-NN-13	KC357730	HC14

JX291990	GX-NN-14	KC357731	HC15
JX291991	GX-NN-15	KC357732	HC16
JX291992	GX-GL11077	KC357733	HC17
JX291993	GX-GL11078	KC414154	chicken/CH/SD/2011/08
JX291994	GX-GL11079	KC346975	SC1201
JX291995	GX-YL11072	JQ900089	ck/CH/JS/2009/1
JX291996	GX-YL11073	JQ900090	ck/CH/JS/2009/2
JX291997	GX-NN11033	JQ900091	ck/CH/JS/2009/3
JX291998	GX-GL2	JQ900092	ck/CH/JS/2009/4
JX291999	GX-NN11034	JQ900093	ck/CH/JS/2009/5
JX292000	GX-NN1009	JQ900094	ck/CH/AH/2009/1
JX292001	GX-NN1011	JQ900095	ck/CH/AH/2009/2
JX292002	GX-NN1012	JQ900096	ck/CH/AH/2009/3
JX292003	GX-NN1013	JQ900097	ck/CH/FJ/2009/1
JX292004	GX-NN1014	JQ900098	ck/CH/GX/2009/1
JX292005	GX-NN1019	JQ900099	ck/CH/JL/2009/1
JX292006	GX-YL1121	JQ900100	ck/CH/AH/2010/5
JX292007	GD-KP1033	JQ900101	ck/CH/JS/2010/1
JX292008	GX-GL	JQ900102	ck/CH/JS/2010/2
JX292009	GX-HC1006	JQ900103	ck/CH/SD/2010/1
JX292010	GX-YL1002	JQ900104	ck/CH/JL/2010/1
JX292011	GX-NN09093	JQ900105	ck/CH/ZJ/2010/2
JX292012	GX-QZ09094	JQ900106	ck/CH/JS/2010/3
JX292013	GX-NN09032	JQ900107	ck/CH/AH/2010/7
JX292014	GX-YL0902	JQ900108	ck/CH/AH/2010/8
KC478590	SC1204	JQ900109	ck/CH/ZJ/2010/3
KC478591	SC1203	JQ900110	ck/CH/AH/2010/9
KC478592	SC1202	JQ900111	ck/CH/AH/2010/11
KC430863	GX-YL4	JQ900112	ck/CH/JS/2010/6
KC357716	HC18	JQ900113	ck/CH/JS/2010/7
KC357717	HC1	JQ900114	ck/CH/JS/2010/8
KC357718	HC2	JQ900115	ck/CH/JS/2010/9
KC357719	HC3	JQ900116	ck/CH/ZJ/2010/4
KC357720	HC4	JQ900117	ck/CH/ZJ/2010/5
KC357721	HC5	JQ900118	ck/CH/JL/2010/2
KC357722	HC6	JQ900119	ck/CH/AH/2010/13
KC357724	HC8	JQ900120	ck/CH/AH/2010/14
KC357725	HC9	JQ900121	ck/CH/JL/2010/3
KC357726	HC10	JQ900122	ck/CH/JS/2010/12
KC357727	HC11	JQ900123	ck/CH/JS/2010/13
KC357728	HC12	JQ900124	ck/CH/ZJ/2010/6
JQ900129	ck/CH/JS/2011/7	JQ900125	ck/CH/ZJ/2010/7
JQ900130	ck/CH/JS/2011/8	JQ900126	ck/CH/JS/2011/2
JQ900131	ck/CH/AH/2011/2	JQ900127	ck/CH/JS/2011/5

JQ900132	ck/CH/JL/2011/1	JQ900128	ck/CH/JS/2011/6
JQ900133	ck/CH/HeB/2011/1	JQ739257	ck/CH/LHB/110957
JQ900134	ck/CH/JS/2011/9	JQ739258	ck/CH/LHB/110968
JQ900135	ck/CH/JS/2011/10	JQ739259	ck/CH/LHB/111101
JQ900136	ck/CH/SD/2011/1	JQ739260	ck/CH/LHB/111102
JQ900137	ck/CH/AH/2011/3	JQ739261	ck/CH/LHB/111107
JQ900138	ck/CH/HeB/2011/2	JQ739262	ck/CH/LHB/111108
JQ900139	ck/CH/JS/2011/11	JQ739263	ck/CH/LHB/111109
JQ900140	ck/CH/JS/2011/12	JQ739264	ck/CH/LHB/111116
JQ900141	ck/CH/JS/2011/13	JQ739265	ck/CH/LHB/111124
JX569792	ck/CH/HN/1205	JQ739266	ck/CH/LHB/111127
JX535283	SCTW	JQ739267	ck/CH/LHB/111128
JQ234966	GX-GL1121	JQ739268	ck/CH/LHB/111146
JX436328	GX-GL1	JQ739269	ck/CH/LHB/111148
JX436329	GX-NN4	JQ739270	ck/CH/LHB/111149
JX436330	GX-GL2005	JQ739271	ck/CH/LHB/111167
JX436331	GX-NN1201	JQ739272	ck/CH/LHB/111168
JX003634	GX-LC1124	JQ739273	ck/CH/LHB/111172
JQ739235	ck/CH/LAH/111115	JQ739274	ck/CH/LHB/111173
JQ739236	ck/CH/LDL/110671	JQ739275	ck/CH/LHB/111184
JQ739237	ck/CH/LDL/110931	JQ739276	ck/CH/LHB/111190
JQ739238	ck/CH/LHB/110121	JQ739277	ck/CH/LHB/111192
JQ739239	ck/CH/LHB/110123	JQ739278	ck/CH/LHB/111207
JQ739240	ck/CH/LHB/110526	JQ739279	ck/CH/LHB/111211
JQ739241	ck/CH/LHB/110605	JQ739280	ck/CH/LHB/111224
JQ739242	ck/CH/LHB/110607	JQ739281	ck/CH/LHB/111225
JQ739243	ck/CH/LHB/110615	JQ739282	ck/CH/LHB/111227
JQ739244	ck/CH/LHB/110617	JQ739283	ck/CH/LHB/111231
JQ739245	ck/CH/LHB/110661	JQ739284	ck/CH/LHB/111232
JQ739246	ck/CH/LHB/110753	JQ739285	ck/CH/LHB/111254
JQ739247	ck/CH/LHB/110754	JQ739286	ck/CH/LHB/111256
JQ739248	ck/CH/LHB/110803	JQ739287	ck/CH/LHB/111258
JQ739249	ck/CH/LHB/110806	JQ739288	ck/CH/LHB/111265
JQ739250	ck/CH/LHB/110816	JQ739289	ck/CH/LHB/111267
JQ739251	ck/CH/LHB/110817	JQ739290	ck/CH/LHB/111268
JQ739252	ck/CH/LHB/110818	JQ739291	ck/CH/LHB/1112103
JQ739253	ck/CH/LHB/110820	JQ739292	ck/CH/LHB/1112104
JQ739254	ck/CH/LHB/110825	JQ739293	ck/CH/LHB/1112109
JQ739255	ck/CH/LHB/110846	JQ739294	ck/CH/LHLJ/110310
JQ739256	ck/CH/LHB/110849	JQ739295	ck/CH/LHLJ/110530
JQ739296	ck/CH/LHLJ/110620	JQ739339	ck/CH/LSD/110115
JQ739297	ck/CH/LHLJ/110621	JQ739340	ck/CH/LSD/110311
JQ739298	ck/CH/LHLJ/110658	JQ739341	ck/CH/LSD/110312
JQ739299	ck/CH/LHLJ/110664	JQ739342	ck/CH/LSD/110314

JQ739300	ck/CH/LHLJ/110672	JQ739343	ck/CH/LSD/110315
JQ739301	ck/CH/LHLJ/110673	JQ739344	ck/CH/LSD/110325
JQ739302	ck/CH/LHLJ/110772	JQ739345	ck/CH/LSD/110326
JQ739303	ck/CH/LHLJ/110835	JQ739346	ck/CH/LSD/110328
JQ739304	ck/CH/LHLJ/110836	JQ739347	ck/CH/LSD/110330
JQ739305	ck/CH/LHLJ/110901	JQ739348	ck/CH/LSD/110334
JQ739306	ck/CH/LHLJ/110902	JQ739349	ck/CH/LSD/110347
JQ739307	ck/CH/LHLJ/110907	JQ739350	ck/CH/LSD/110348
JQ739308	ck/CH/LHLJ/110909	JQ739351	ck/CH/LSD/110408
JQ739309	ck/CH/LHLJ/110935	JQ739352	ck/CH/LSD/110409
JQ739310	ck/CH/LHLJ/110943	JQ739353	ck/CH/LSD/110410
JQ739311	ck/CH/LHLJ/110949	JQ739354	ck/CH/LSD/110415
JQ739312	ck/CH/LHLJ/110966	JQ739355	ck/CH/LSD/110417
JQ739313	ck/CH/LHLJ/111023	JQ739356	ck/CH/LSD/110435
JQ739314	ck/CH/LHLJ/111043	JQ739357	ck/CH/LSD/110505
JQ739315	ck/CH/LHLJ/111050	JQ739358	ck/CH/LSD/110510
JQ739316	ck/CH/LHLJ/111119	JQ739359	ck/CH/LSD/110511
JQ739317	ck/CH/LHLJ/111154	JQ739360	ck/CH/LSD/110520
JQ739318	ck/CH/LHLJ/111205	JQ739361	ck/CH/LSD/110529
JQ739319	ck/CH/LHLJ/111246	JQ739362	ck/CH/LSD/110707
JQ739320	ck/CH/LHN/110352	JQ739363	ck/CH/LSD/110712
JQ739321	ck/CH/LHN/110518	JQ739364	ck/CH/LSD/110719
JQ739322	ck/CH/LHN/111283	JQ739365	ck/CH/LSD/110722
JQ739323	ck/CH/LJL/110302	JQ739366	ck/CH/LSD/110723
JQ739324	ck/CH/LJL/110303	JQ739367	ck/CH/LSD/110726
JQ739325	ck/CH/LJL/110437	JQ739368	ck/CH/LSD/110731
JQ739326	ck/CH/LJL/110439	JQ739369	ck/CH/LSD/110733
JQ739327	ck/CH/LJL/110654	JQ739370	ck/CH/LSD/110739
JQ739328	ck/CH/LJL/111052	JQ739371	ck/CH/LSD/110741
JQ739329	ck/CH/LJL/111054	JQ739372	ck/CH/LSD/110851
JQ739330	ck/CH/LJS/111111	JQ739373	ck/CH/LSD/110854
JQ739331	ck/CH/LJS/111208	JQ739374	ck/CH/LSD/110856
JQ739332	ck/CH/LJS/111209	JQ739375	ck/CH/LSD/110857
JQ739333	ck/CH/LJS/111210	JQ739376	ck/CH/LSD/110910
JQ739334	ck/CH/LLN/111169	JQ739377	ck/CH/LSD/110912
JQ739335	ck/CH/LNM/110932	JQ739378	ck/CH/LSD/110913
JQ739336	ck/CH/LNM/110943	JQ739379	ck/CH/LSD/110918
JQ739337	ck/CH/LSD/110112	JQ739380	ck/CH/LSD/111025
JQ739338	ck/CH/LSD/110113	JQ739381	ck/CH/LSD/111037
JQ739382	ck/CH/LSD/111041	JF951374	SCMS-1
JQ739383	ck/CH/LSD/111042	JF951375	SCMS-2
JQ739384	ck/CH/LSD/111045	JF951376	SCMS-3
JQ739385	ck/CH/LSD/111218	JF951377	SCMS-4
JQ739386	ck/CH/LSD/111219	JF738084	ck/CH/SD10/002

JQ739387	ck/CH/LSD/111220	JF738085	ck/CH/SD10/003
JQ739388	ck/CH/LSD/111221	JF330848	ck/CH/LDL/101212
JQ739389	ck/CH/LSD/111222	JF330849	ck/CH/LDL/101215
JQ739390	ck/CH/LSD/111223	JF330850	ck/CH/LGS/100521
JQ739391	ck/CH/LSD/111235	JF330851	ck/CH/LHB/100904
JQ739392	ck/CH/LSD/111241	JF330852	ck/CH/LHB/100906
JQ739393	ck/CH/LSD/111261	JF330853	ck/CH/LHB/100908
JQ739394	ck/CH/LSD/111266	JF330854	ck/CH/LHLJ/100309
JQ739395	ck/CH/LSD/111297	JF330855	ck/CH/LHLJ/100902
JQ739396	ck/CH/LSD/1112114	JF330856	ck/CH/LHN/101208
JQ739397	ck/CH/LSD/1112115	JF330857	ck/CH/LHN/101209
JQ739398	ck/CH/LSD/1112130	JF330858	ck/CH/LHN/101210
JQ739399	ck/CH/LSD/1112132	JF330859	ck/CH/LHN/101211
JQ739400	ck/CH/LSD/1112133	JF330860	ck/CH/LJL/100501
JQ739401	ck/CH/LSD/1112134	JF330861	ck/CH/LJL/100512
JQ739402	ck/CH/LSD/1112138	JF330862	ck/CH/LJL/100513
JQ739403	ck/CH/LSD/1112139	JF330863	ck/CH/LJL/100515
JQ739404	ck/CH/LSD/1112142	JF330864	ck/CH/LJL/100713
JQ739405	ck/CH/LSD/1112147	JF330865	ck/CH/LJL/100918
JQ739406	ck/CH/LSD/1112150	JF330866	ck/CH/LJL/101131
JQ739407	ck/CH/LSD/1112155	JF330867	ck/CH/LJS/101105
JQ739408	ck/CH/LXJ/111265	JF330868	ck/CH/LJL/101150
JQ739409	ck/CH/LZJ/111113	JF330869	ck/CH/LJS/101106
JQ739410	ck/CH/LZJ/111114	JF330870	ck/CH/LJS/101108
JQ801378	HH11	JF330871	ck/CH/LJS/101109
JQ250818	QS	JF330872	ck/CH/LJS/101110
JN032761	ck/CH/AH/2010/II	JF330873	ck/CH/LJS/101111
JF682372	XT	JF330874	ck/CH/LJS/101112
JF694495	JS	JF330875	ck/CH/LJS/101113
JF694496	HP	JF330876	ck/CH/LJS/101114
JF694497	JZ	JF330877	ck/CH/LJS/101115
JF951367	SCCQ-1	JF330878	ck/CH/LJS/101237
JF951368	SCZJ-1	JF330879	ck/CH/LLN/101213
JF951369	SCZJ-2	JF330880	ck/CH/LSD/100102
JF951370	SCZJ-3	JF330881	ck/CH/LSD/100205
JF951371	SCZJ-4	JF330882	ck/CH/LSD/100208
JF951372	SCDY-1	JF330883	ck/CH/LSD/100305
JF951373	SCDY-2	JF330884	ck/CH/LSD/100311
JF330885	ck/CH/LSD/100312	HQ018905	ck/CH/GX/YL09-2
JF330886	ck/CH/LSD/100315	HQ018906	ck/CH/GD/XX10
JF330887	ck/CH/LSD/100318	HQ018907	ck/CH/GD/XX09
JF330888	ck/CH/LSD/100504	HQ018908	ck/CH/GD/YN09-1
JF330889	ck/CH/LSD/100505	HQ018909	ck/CH/GD/YN09-2
JF330890	ck/CH/LSD/101139	HQ018910	ck/CH/GX/GL10

JF330891	ck/CH/LSD/101216	HQ018911	ck/CH/GX/LC10
JF330892	ck/CH/LSD/101217	HQ018912	ck/CH/GX/YL10-1
JF330893	ck/CH/LSD/101221	HQ018913	ck/CH/GX/YL10-2
JF330894	ck/CH/LSD/101222	HQ018914	ck/CH/SC/MS10
JF330895	ck/CH/LSD/101223	HQ018915	ck/CH/HN/HN10
JF330896	ck/CH/LSD/101224	HQ018916	ck/CH/HeB/CZ10
JF330897	ck/CH/LSD/101225	HQ018917	ck/CH/TJ/NH10
JF330900	ck/CH/LJS/101116	HQ018918	ck/CH/SC/ZJ10-1
JF330901	ck/CH/LJS/101117	HQ018919	ck/CH/GD/KP10
JF330902	ck/CH/LSD/100408	HQ018920	ck/CH/SC/ZJ10-2
JF330903	ck/CH/LSD/100409	HQ018921	ck/CH/GD/ZX10
JF330904	ck/CH/LSD/100412	HM540074	GX-NN2
HQ132760	GX-NN1	HM540075	GX-NN3
HQ185567	ck/TW/T15/2006	HM540076	GX-NN9
HQ018882	ck/CH/SC/DY09	HM540077	GX-NN11
HQ018883	ck/CH/HB/HC09-1	HM540078	GX-YL3
HQ018884	ck/CH/HB/HC09-2	HQ018928	GX-YL8
HQ018885	ck/CH/AH/HF10	HQ398360	0801
HQ018886	ck/CH/HN/HN09	HM230749	ck/CH/SD09/005
HQ018887	ck/CH/GD/HY09	HM194639	ck/CH/LDL/091021
HQ018888	ck/CH/ZJ/HZ09	HM194640	ck/CH/LDL/091022
HQ018889	ck/CH/ZJ/HZ10	HM194641	ck/CH/LGD/090907
HQ018890	ck/CH/JX/JA09-1	HM194642	ck/CH/LGX/091109
HQ018891	ck/CH/JX/JA09-2	HM194643	ck/CH/LGX/091110
HQ018892	ck/CH/HB/JL09	HM194644	ck/CH/LGX/091111
HQ018893	ck/CH/JS/JL10	HM194645	ck/CH/LGX/091112
HQ018894	ck/CH/GD/LY10	HM194646	ck/CH/LHB/090404
HQ018895	ck/CH/JS/LYG09	HM194647	ck/CH/LHB/090406
HQ018896	ck/CH/GD/LZ09	HM194648	ck/CH/LHB/090914
HQ018897	ck/CH/SC/MS09	HM194649	ck/CH/LHB/090916
HQ018898	ck/CH/FJ/PT10	HM194650	ck/CH/LHB/090919
HQ018899	ck/CH/HuN/NX09	HM194651	ck/CH/LHB/090920
HQ018900	ck/CH/GX/NN09	HM194652	ck/CH/LHB/090921
HQ018901	ck/CH/JS/NJ10	HM194653	ck/CH/LHB/090924
HQ018902	ck/CH/JS/NJ09	HM194654	ck/CH/LHLJ/090323
HQ018903	ck/CH/GD/NC10	HM194655	ck/CH/LHLJ/090410
HQ018904	ck/CH/GX/YL09-1	HM194656	ck/CH/LHLJ/090428
HM194657	ck/CH/LHLJ/090438	GU938365	ck/CH/Guangdong/Xinnong/0811
HM194658	ck/CH/LHLJ/090510	GU938366	ck/CH/Guangdong/Lianhua/0806
HM194659	ck/CH/LHLJ/090515	GU938367	ck/CH/Guangdong/DashaY9/0902
HM194660	ck/CH/LHLJ/090603	GU938368	ck/CH/Guangdong/DashaY4/0902
HM194661	ck/CH/LHLJ/090604	GU938369	ck/CH/Hubei/Wuhan1/0901
HM194662	ck/CH/LHLJ/090605	GU938370	ck/CH/Hubei/WuHan2/0901
HM194663	ck/CH/LHLJ/090607	GU938371	ck/CH/Hubei/WuHan3/0901

HM194664	ck/CH/LHLJ/090619	GU938372	ck/CH/Hubei/WuHan4/0901
HM194665	ck/CH/LHLJ/090640	GU938373	ck/CH/Guangdong/Xinnong1/0901
HM194666	ck/CH/LHLJ/090712	GU938374	ck/CH/Guangdong/Xinnong2/0901
HM194667	ck/CH/LHLJ/090805	GU938375	ck/CH/Guangdong/Shuitai/0901
HM194668	ck/CH/LHLJ/090806	GU938376	ck/CH/Hainan1/0903
HM194669	ck/CH/LHLJ/090908	GU938377	ck/CH/Hainan2/0903
HM194670	ck/CH/LHLJ/090912	GU938378	ck/CH/Hainan1/0904
HM194671	ck/CH/LHLJ/091202	GU938379	ck/CH/Hainan2/0904
HM194672	ck/CH/LHLJ/091205	GU938380	ck/CH/Hainan3/0904
HM194673	ck/CH/LHN/091025	GU938381	ck/CH/Hainan4/0904
HM194674	ck/CH/LJL/090330	GU938382	ck/CH/Hainan1/0901
HM194675	ck/CH/LJL/090419	GU938383	ck/CH/Hainan2/0901
HM194676	ck/CH/LJL/090608	GU938384	ck/CH/Hainan3/0903
HM194677	ck/CH/LJL/090614	GU938385	ck/CH/Guangdong/Baitu/0904
HM194678	ck/CH/LLN/090312	GU938386	ck/CH/Guangdong/Heyuan1/0904
HM194679	ck/CH/LLN/090907	GU938387	ck/CH/Guangdong/Heyuan2/0904
HM194680	ck/CH/LLN/090909	GU938388	ck/CH/Guangdong/Lezhu3/0905
HM194681	ck/CH/LLN/090910	GU938389	ck/CH/Guangxi/Guilin/0811
HM194682	ck/CH/LNM/091017	GU938390	ck/CH/Guangxi/Guilin1/0806
HM194683	ck/CH/LNX/090442	HM159252	ck/CH/SD09/003
HM194684	ck/CH/LSD/090314	HM159253	ck/CH/SD09/004
HM194685	ck/CH/LSD/090316	HM159254	ck/CH/SD09/006
HM194686	ck/CH/LSD/090320	HM159255	ck/CH/SD09/002
HM194687	ck/CH/LSD/090326	HM113492	FJ09
HM194688	ck/CH/LSD/090334	HM113493	HN09-1
HM194689	ck/CH/LSD/090401	HM113494	HN09-2
HM194690	ck/CH/LSD/090402	HM113495	JX09
HM194691	ck/CH/LSD/090411	HM113496	SS09-1
HM194692	ck/CH/LSD/090432	HM113497	XX09-3
HM194693	ck/CH/LSD/090434	HM106334	ck/CH/SCMS/10I
HM194694	ck/CH/LSD/090437	HM106335	ck/CH/SCCD/10I
HM194695	ck/CH/LSD/090440	HM106336	ck/CH/SCDY/10I
HM194696	ck/CH/LSD/090509	HM034813	YI
HM194697	ck/CH/LSD/090517	HM034814	WZL
HM194698	ck/CH/LSD/090518	HM031117	A2-2
HM194699	ck/CH/LSD/090520	HM031118	S-03
HM194700	ck/CH/LSD/090521	GU938391	ck/CH/Guangxi/Yulin/0812
HM194701	ck/CH/LSD/090708	GU938392	ck/CH/Jiangsu/Lianyungang/0902
HM194702	ck/CH/LSD/090709	GU938393	ck/CH/Guangxi/Yulin/0906
HM194703	ck/CH/LSD/090710	GU938394	ck/CH/Guangxi/Luchuan/0906
HM194704	ck/CH/LSD/090809	GU938395	ck/CH/Guangxi/Hezhou/0903
HM194705	ck/CH/LSD/090816	GU938396	ck/CH/Guangxi/Guilin2/0806
HM194706	ck/CH/LSD/090902	GU938397	ck/CH/Guangdong/Lezhu1/0905
HM194707	ck/CH/LSD/091003	GU938398	ck/CH/Guangdong/Lezhu2/0905

HM194708	ck/CH/LSD/091004	GU938399	ck/CH/Guangxi/Yulin/0904
HM194709	ck/CH/LSD/091005	GU938400	ck/CH/Guangxi/Nanning/0903
HM194710	ck/CH/LSD/091014	GU938401	ck/CH/Guangdong/Heyuan1/0905
HM194711	ck/CH/LSD/091108	GU938402	ck/CH/Guangdong/Keyanjidi/0908
HM194712	ck/CH/LSD/091154	GU938403	ck/CH/Guangxi/Luchuan1/0910
HM194713	ck/CH/LSD/091159	GU938404	ck/CH/Guangxi/Luchuan2/0910
HM194714	ck/CH/LSD/091203	GU938405	ck/CH/Guangxi/Luchuan3/0910
HM194715	ck/CH/LSD/091204	GU938406	ck/CH/Guangdong/Heyuan1/0910
HM194716	ck/CH/LSD/090519	GU938407	ck/CH/Guangdong/Heyuan2/0910
HM363025	ck/CH/SCNC/08I	GU938408	ck/CH/Fujian/Putian1/0910
HM363026	ck/CH/SCSN/10I	GU938409	ck/CH/Fujian/Putian2/0910
HM363027	ck/CH/SCYA/10I	GU938410	ck/CH/Fujian/Putian3/0910
HM363028	ck/CH/SCMY/10I	GU938411	ck/CH/Fujian/Putian4/0910
HM159247	ck/CH/SD10/001	GU938412	ck/CH/NanJiao/0904
HM159248	ck/CH/SD08/006	GU938425	ck/CH/Zhejiang/Huzhou1/0910
HM159249	ck/CH/SD08/007	GU938426	ck/CH/Zhejiang/Huzhou2/0910
HM159250	ck/CH/SD08/008	GU938427	ck/CH/Zhejiang/Huzhou3/0910
HM159251	ck/CH/SD09/001	GU938428	ck/CH/Zhejiang/Huzhou4/0910
GU938413	ck/CH/Chongqing/0908	GU938429	ck/CH/Zhejiang/Huzhou5/0910
GU938414	ck/CH/Chongqing/0909	GU938430	ck/CH/Zhejiang/Huzhou6/0910
GU938415	ck/CH/Sichuan/Meishan/0910	GU938431	ck/CH/Zhejiang/Quzhou1/0910
GU938416	ck/CH/Guangdong/Heyuan2/0905	GU938432	ck/CH/Zhejiang/Quzhou2/0910
GU938417	ck/CH/Guangxi/Luchuan4/0910	GU938433	ck/CH/Zhejiang/Quzhou3/0910
GU938418	ck/CH/Guangxi/Luchuan5/0910	GU938434	ck/CH/Zhejiang/Huzhou7/0910
GU938419	ck/CH/Guangxi/Luchuan6/0910	GU938435	ck/CH/Zhejiang/Huzhou8/0910
GU938420	ck/CH/Guangxi/Luchuan7/0910	GU938436	ck/CH/Zhejiang/Huzhou9/0910
GU938421	ck/CH/Guangdong/Heyuan3/0910	GU938437	ck/CH/Guangdong/Shuitai/0903
GU938422	ck/CH/Guangxi/Luchuan8/0910	GU938438	ck/CH/Guangdong/Xindadi/0902
GU938423	ck/CH/Guangdong/Shalang/0910	GU938439	ck/CH/Hainan/0811
GU938424	ck/CH/Guangxi/Luchuan9/0910	GU938440	ck/CH/Guangdong/Wulian/0901
GU938441	ck/CH/Guangdong/Fengmulang/0901	GQ258307	ck/CH/LDL/08II
GU938442	ck/CH/Guangdong/Xindadi/0903	GQ258308	ck/CH/LDL/08III
GU938443	ck/CH/Guangdong/Heyuan/0902	GQ258309	ck/CH/LDL/08IV
GU938444	ck/CH/Hainan4/0903	GQ258310	ck/CH/LGS/08I
GU384207	ck/CH/EM/09I	GQ258311	ck/CH/LHB/08I
GU727853	ck/CH/SCBY/09I	GQ258312	ck/CH/LHLJ/08I
GU727854	ck/CH/CD/08I	GQ258313	ck/CH/LHLJ/08II
GU727855	ck/CH/SCCD/09I	GQ258314	ck/CH/LHLJ/08III
GU727856	ck/CH/SCDY/09I	GQ258315	ck/CH/LHLJ/08IV
GU727857	ck/CH/SCLS/08I	GQ258316	ck/CH/LJL/08I
GU727858	ck/CH/SCMS/08I	GQ258317	ck/CH/LJL/08II
GU727859	ck/CH/SCMS/09I	GQ258318	ck/CH/LJL/08III
GU727860	ck/CH/SCMY/09I	GQ258319	ck/CH/LJL/08IV
GU727861	ck/CH/SCYA/08I	GQ258320	ck/CH/LJS/08I

GU727862	ck/CH/SCZG/08I	GQ258321	ck/CH/LJS/08II
GU455379	Jin-13	GQ258322	ck/CH/LLN/08I
GU455380	XP/1/09	GQ258323	ck/CH/LLN/08II
GU455381	XP/3	GQ258324	ck/CH/LSD/08-1
GQ853588	IBVSX7	GQ258325	ck/CH/LSD/08-10
GU455384	IBVSX9	GQ258326	ck/CH/LSD/08-11
GQ853590	IBVSX5	GQ258327	ck/CH/LSD/08-12
GQ844985	Beijing-98	GQ258328	ck/CH/LSD/08-2
GQ844986	Chongqing-06	GQ258329	ck/CH/LSD/08-3
GQ844987	Deyang-06	GQ258330	ck/CH/LSD/08-4
GQ844988	Guangzhou-06	GQ258331	ck/CH/LSD/08-5
GQ844989	Guilin-06	GQ258332	ck/CH/LSD/08-6
GQ844990	KD-08	GQ258333	ck/CH/LSD/08-7
GQ844991	Sichuan-06	GQ258334	ck/CH/LSD/08-8
GQ844992	Taian-03	GQ258335	ck/CH/LSD/08-9
GQ258302	ck/CH/LAH/08I	GQ258336	ck/CH/LSD/08I
GQ258303	ck/CH/LAH/08II	GQ258337	ck/CH/LSD/08II
GQ258304	ck/CH/LCQ/08I	GQ258338	ck/CH/LSD/08III
GQ258305	ck/CH/LCQ/08II	GQ258339	ck/CH/LSD/08IV
GQ258306	ck/CH/LDL/08I	GQ258340	ck/CH/LSD/08IX
GQ229240	3370/05	GQ258341	ck/CH/LSD/08V
GQ229241	3371/05	GQ258342	ck/CH/LSD/08VI
GQ229242	3372/05	GQ258343	ck/CH/LSD/08VII
GQ229243	3373/05	GQ258344	ck/CH/LSD/08VIII
GQ229244	3376/06	GQ258345	ck/CH/LSD/08X
GQ229245	3381/06	GQ258346	ck/CH/LSD/08XI
GQ229246	3384/06	GQ258347	ck/CH/LSD/08XII
GQ229247	3385/06	GQ229237	3339/05
GQ265927	DY07	GQ229238	3368/05
GQ265928	DY05	GQ229239	3369/05
GQ265929	FS08-1	FJ599752	HN/HL
GQ265930	FS08-2	FJ426564	SDZB0807
GQ265931	GM05	FJ426565	SDZB0808
GQ265932	GL08-1	FJ345364	ck/CH/LDL/07I
GQ265933	GL08-2	FJ345365	ck/CH/LDL/07III
GQ265934	HB08	FJ345366	ck/CH/LHLJ/07III
GQ265935	SS06-1	FJ345367	ck/CH/LHLJ/07IV
GQ265936	SS07	FJ345368	ck/CH/LHLJ/07VI
GQ265937	SS06-2	FJ345369	ck/CH/LHLJ/07VII
GQ265938	HN06-1	FJ345370	ck/CH/LHN/07I
GQ265939	HN06-2	FJ345371	ck/CH/LHN/07II
GQ265940	HN08	FJ345372	ck/CH/LHN/07III
GQ265941	HY06	FJ345373	ck/CH/LJL/07I
GQ265942	HY07	FJ345374	ck/CH/LJL/07II

GQ265943	LZ05	FJ345375	ck/CH/LJL/07III
GQ265944	LZ07	FJ345376	ck/CH/LJS/07I
GQ265945	XX08	FJ345377	ck/CH/LJS/07II
GQ265946	NN07	FJ345378	ck/CH/LJS/07IV
GQ265947	TC07-1	FJ345379	ck/CH/LJS/07V
GQ265948	TC07-2	FJ345380	ck/CH/LLN/07I
GQ265949	ZX07	FJ345381	ck/CH/LSD/07-1
GQ265950	DY04	FJ345382	ck/CH/LSD/07-2
GQ265951	NN04	FJ345383	ck/CH/LSD/07-3
GQ265952	CQ04-1	FJ345384	ck/CH/LSD/07II
GQ265953	CQ04-2	FJ345385	ck/CH/LSD/07III
GQ154654	HN/SG	FJ345386	ck/CH/LSD/07IV
GQ154655	H120	FJ345387	ck/CH/LSD/07IX
FJ907238	GX-YL5	FJ345388	ck/CH/LSD/07V
FJ907239	GX-NN7	FJ210647	SDZB0804
FJ793938	IBVSX2	EU930426	SDSG0801
FJ793939	IBVSX4	EU930427	SDTA06112
FJ793940	IBVSX8	EU930428	SDWF0711
FJ793941	IBVSX16	EU930429	SDWF0608
EU930430	SDLY0702	DQ459475	BJ03-1
EU930431	SDZB0803	DQ459476	CQ04-1
EU930432	SDZB0708	DQ402364	3374/05
EU930433	SDPY0703	AY772944	BJ9601
EU930434	SDLY0701	DQ167128	ck/CH/LAH/03I
EU930435	SDTA06111	DQ167129	ck/CH/LAH/99I
EU930436	SDYT0605	DQ167130	ck/CH/LDL/01I
EU930437	SDJY0701	DQ167131	ck/CH/LDL/04II
EU031525	ck/CH/JS/06I	DQ167132	ck/CH/LDL/98I
EU031526	ck/CH/JS/06II	DQ167133	ck/CH/LGD/03I
EU031527	ck/CH/JS/06III	DQ167134	ck/CH/LGD/04II
EU031528	ck/CH/JS/07I	DQ167135	ck/CH/LGD/04III
EU031529	ck/CH/JS/07II	DQ167136	ck/CH/LGD/96I
EU031530	ck/CH/JS/07III	DQ167137	ck/CH/LHB/96I
EU031531	ck/CH/JS/07V	DQ167138	ck/CH/LHLJ/02I
EU857816	SDLY0612	DQ167139	ck/CH/LHLJ/04V
EU650418	XG	DQ167140	ck/CH/LHLJ/04XI
EU563940	ck/CH/LDL/07II	DQ167141	ck/CH/LHLJ/95I
EU563941	ck/CH/LSD/07I	DQ167142	ck/CH/LHLJ/99I
EU563942	ck/CH/LHLJ/07I	DQ167143	ck/CH/LHN/00I
EU563943	ck/CH/LHLJ/07V	DQ167144	ck/CH/LJL/04I
EU563944	ck/CH/LJL/07V	DQ167145	ck/CH/LLN/98I
EF577030	HH06	DQ167146	ck/CH/LSC/95I
EF213558	ck/CH/LDL05/III	DQ167147	ck/CH/LSC/99I
EF213559	ck/CH/LDL/05II	DQ167148	ck/CH/LSD/03I

EF213560	ck/CH/LHLJ/05I	DQ167149	ck/CH/LSHH/03I
EF213561	ck/CH/LHLJ/06II 2008	DQ167150	ck/CH/LSHH/03II
EF213562	ck/CH/LJL/05I	DQ167151	ck/CH/LTJ/95I
EF213563	ck/CH/LDL/05I	DQ167152	ck/CH/LXJ/02I
EF213564	ck/CH/LGS/06I	DQ075323	SH1
EF213565	ck/CH/LHLJ/05VI	DQ075324	SH2
EF213566	ck/CH/LLN/06I	DQ072273	SH5
EF213567	ck/CH/LNM/05I	DQ072274	SH6
EF213568	ck/CH/LSD/05I	DQ069317	SH3
EF213580	ck/CH/LGX/06I	DQ069318	SH4
EF030996	ck/CH/LDL/97I	AY837465	TA03
AB274268	TX94a	AY775551	HN99
AB274269	TX94b	AY769993	CQ/01/2004
AB274270	TX95	AY702970	HN9604
AB274271	DB03	AY606314	2575/98
DQ459472	SC03-1	AY606315	T03/01
DQ459473	GD04-1	AY606316	2993/02
DQ459474	YN05-01	AY606317	3025/02
AY606318	3051/02	AF488350	D971
AY606319	3071/03	AF395743	1/98
AY606320	TP/64	AF395744	2/97
AY606321	2296/95	AF395745	3/97
AY606322	T07/02	AY043221	X
AY606323	2992/02	AY043312	A2
AY606324	2994/02	AF352313	ZJ971
AY427818	W93	AF250005	A1171
AY296742	TW97-4	AF274435	DE072
AY319302	GX1-98	AF250006	A1211
AY277632	LD3	AF208239	JS/95/03
AY278246	LS2	AF208240	SD/97/01
AY251816	GX2-98	AF210735	JX/99/01
AY251817	HaN1-95	AY237817	SC021202
